# Effects of the social environment during adolescence on the development of social behaviour, hormones and morphology in male zebra finches (*Taeniopygia guttata*)

**DOI:** 10.1186/s12983-017-0190-4

**Published:** 2017-01-25

**Authors:** Stefanie Bölting, Nikolaus von Engelhardt

**Affiliations:** 1 Department of Animal Behaviour, Bielefeld University, 33615 Bielefeld, Germany; 2 Faculty of Science and Engineering, University of Plymouth, Drake Circus, Plymouth, PL4 8AA UK

**Keywords:** Social environment, Adolescence, Social interactions, Maturation, Testosterone, Corticosterone, Plumage colouration, Courtship, Aggression, Song

## Abstract

**Background:**

Individual differences in behaviour are widespread in the animal kingdom and often influenced by the size or composition of the social group during early development. In many vertebrates the effects of social interactions early in life on adult behaviour are mediated by changes in maturation and physiology. Specifically, increases in androgens and glucocorticoids in response to social stimulation seem to play a prominent role in shaping behaviour during development. In addition to the prenatal and early postnatal phase, adolescence has more recently been identified as an important period during which adult behaviour and physiology are shaped by the social environment, which so far has been studied mostly in mammals. We raised zebra finches (*Taeniopygia guttata*) under three environmental conditions differing in social complexity during adolescence - juvenile pairs, juvenile groups, and mixed-age groups - and studied males’ behavioural, endocrine, and morphological maturation, and later their adult behaviour.

**Results:**

As expected, group-housed males exhibited higher frequencies of social interactions. Group housing also enhanced song during adolescence, plumage development, and the frequency and intensity of adult courtship and aggression. Some traits, however, were affected more in juvenile groups and others in mixed-age groups. Furthermore, a testosterone peak during late adolescence was suppressed in groups with adults. In contrast, corticosterone concentrations did not differ between rearing environments. Unexpectedly, adult courtship in a test situation was lowest in pair-reared males and aggression depended upon the treatment of the opponent with highest rates shown by group-reared males towards pair-reared males. This contrasts with previous findings, possibly due to differences in photoperiod and the acoustic environment.

**Conclusion:**

Our results support the idea that effects of the adolescent social environment on adult behaviour in vertebrates are mediated by changes in social interactions affecting behavioural and morphological maturation. We found no evidence that long-lasting differences in behaviour reflect testosterone or corticosterone levels during adolescence, although differences between juvenile and mixed-age groups suggest that testosterone and song behaviour during late adolescence may be associated.

## Background

In many species, the social environment during ontogeny [1–3] influences the development of adult social behaviour [4–9]. However, the importance of social experiences for adult behaviour has mostly been demonstrated for the prenatal and early postnatal phase (e.g. [10–12]), and only more recently has evidence accumulated that the social environment during adolescence is also crucial [2, 13, 14]. Adolescence can be defined as the gradual transition from childhood to adulthood [15] and is characterised by marked neuronal, endocrine, morphological and behavioural changes [15–19]. Increases in sex steroids produced by the hypothalamo-pituitary-gonadal (HPG) axis during adolescence are critical in the development and regulation of the reproductive system, adult morphology, reproductive behaviour and sexual maturity [16, 20–23]. In addition, increases in glucocorticoid hormones produced by the hypothalamo-pituitary-adrenal (HPA) axis in response to new stimuli and stressors [2, 19], but see [24] may be important when new environments or unfamiliar conspecifics are encountered during adolescence. Importantly, sex steroid and glucocorticoid levels are affected by social experiences [25–28]. This suggests that variation in social experiences during adolescence may have long-lasting behavioural and morphological consequences via organisational effects of these hormones.

During adolescence, the social environment of juveniles changes considerably in many species as they gain independence from their parents and increasingly interact with other adults and peers [15, 29]. Although these interactions may be stressful, as indicated by increased glucocorticoid levels [2, 30], they allow juveniles to practice important behaviour which they will need in adulthood to reproduce successfully, such as courtship and aggression [1, 31, 32]. The size, age and sex composition of groups affect the amount and type of social interactions juveniles experience, and these are thought to affect future behaviour through learning and neuroendocrine changes [33–35]. Variation in social interactions during adolescence may thus adaptively adjust reproductive behaviour to the social conditions that juveniles are likely to encounter as adults. Studies in a variety of species, e.g. brown-headed cowbirds (*Molothrus ater*), [1, 36, 37], daffodil cichlids (*Neolamprologus pulcher*) [5], guinea pigs (*Cavia apera* f. *porcellus*) [2, 14] and zebra finches (*Taeniopygia guttata*) [3, 38] suggest that a more complex early social environment improves adult social competence.

The most comprehensive studies on the effects of the social environment during adolescence on social interactions, hormones and adult behaviour have been conducted in guinea pigs (*Cavia apera* f. *porcellus*) [2, 30, 39–41]. Guinea pigs are polygynous rodents, in which dominant males aggressively monopolise and court all available females at low social densities, whereas at high social densities males tolerate each other and do not court females bonded to another male. The studies found that males growing up in a mixed-sex group with adults had significantly more social interactions and higher testosterone (T) and cortisol concentrations during adolescence than males growing up in a mixed-sex pair with a female peer. As adults, group-reared males, compared to pair-reared males, showed a lower cortisol response and less aggressive behaviour in an encounter with an unfamiliar male in a new environment and less courtship behaviour towards unfamiliar females [2, 42]. The researchers suggested this reflects an adaptive “queuing strategy” [2]. The authors proposed the following mechanisms by which the adolescent social environment causes adult behavioural modifications: A high frequency of social interactions during adolescence increases T concentrations which reduces the adult cortisol responsiveness that controls the display of adult aggressive behaviour via organisational effects [2].

Interestingly, in zebra finches, the social environment during adolescence has very similar effects on adult courtship and aggression as in guinea pigs. Zebra finch males housed in mixed-sex juvenile groups during adolescence showed less courtship and aggression towards unfamiliar conspecifics in a test situation as adults than males which grew up in mixed-sex juvenile pairs [43]. It is not known, however, whether the behavioural and hormonal changes during adolescence are also similar to guinea pigs, which is the focus of the current study. Zebra finches are highly social, monogamous birds that typically live in large colonies of varying sizes [29]. In these colonies individuals often clump and allopreen, and intense aggression and territoriality normally only occur during breeding [29]. Breeding density can vary considerably between colonies and between individuals. Although most pairs breed within the main colony, others build their nests away from the main colony [29, 44]. The causes of this variation in sociality during breeding are not known.

Despite the lack of direct evidence, there are some indications that similar mechanisms might shape behaviour during adolescence in social birds as in social mammals. In zebra finches, the adolescent period starts around post hatching day 35–40, at full nutritional independence. Sexual maturity is reached around day 60–90, yet morphological, behavioural and physiological maturation continues after sexual maturity [29]. Hence studies often use day 100–110 as the end of adolescence [22, 43]. When reared in isolation from day 31 to day 90 of age, zebra finch males show a significant delay in the development of adult sexual plumage traits compared to socially reared controls [45]. Furthermore, rearing in social deprivation delays song development of zebra finch males [46, 47]. In socially housed individuals, the start of the moult into the adult plumage occurs around day 35, together with the change in beak colour from black to red and a sensitive period for song learning associated with a peak in T [48, 49]. This suggests that social stimulation may lead to developmental peaks in T or other hormones of the HPG axis which may then cause changes in morphological and behavioural transitions. As far as we know, there is no direct evidence yet for effects of social experiences during adolescence on developmental profiles of T.

It is also unclear whether the social environment during adolescence has similar effects on corticosterone (CORT) levels in zebra finches as in guinea pigs [2]. Social isolation of adult zebra finches results in increased CORT levels and reduced vocal activity [50]. Moreover, experimental increases in CORT during early development have negative effects on adult courtship song [51], suggesting that the social environment during adolescence may affect CORT with consequences for adult courtship behaviour.

To understand how the social environment during adolescence may affect adult behaviour in social birds, we experimentally investigated the behavioural, hormonal and morphological changes during adolescence and the resulting adult courtship and aggressive behaviour in male zebra finches by rearing them in three environments differing in social complexity. Earlier studies in zebra finches compared pairs and groups of juveniles and found similar effects on adult behaviour as studies in male guinea pigs using groups comprising both juveniles and adults [2, 43]. Therefore and because in nature zebra finches also grow up with adults present in their environment [29] we also wanted to study whether the presence of adults during adolescence modifies adult behaviour in zebra finches. We housed males in mixed-sex juvenile pairs (one juvenile male and one juvenile female; 1 m/1f), mixed-sex juvenile groups (three juvenile males and three juvenile females; 3 m/3 f), and mixed-sex, mixed-age groups (three juvenile males, three juvenile females, two adult males and two adult females; 5 m/5 f) to study the effects of group housing and the presence of adults in groups on social interactions and hormones during development, and later on adult traits.

Based on the studies in guinea pigs and earlier findings in zebra finches, we predicted that group housing would lead to increased social interactions and elevated or earlier peaks of T and CORT during adolescence. We also predicted that housing males in groups would lead to an accelerated development of song and plumage colouration. Finally, we predicted that group-reared males would show less courtship and aggressive behaviour as adults. Furthermore, we expected that the observed effects might differ between juvenile groups and mixed-age groups.

## Methods

### Subjects and housing conditions

The experimental subjects were initially 50 male domesticated zebra finches *(Taeniopygia guttata),* sired by 22 different breeding pairs at the University of Bielefeld. All males hatched in one of four aviaries located in the same indoor room with a controlled 14 h light:10 h darkness photoperiod (lights on at 7:00 h) with additional natural light entering through windows. Until adolescence, males lived together with their parents, peers and other adults and their offspring. Birds had ad libitum access to standard seed food (Elles, Mischfutter für Exoten, L. Stroetmann Saat, 48163 Münster, Germany) and water. Twice a week, this standard diet was enriched by egg food (Cédé N.V., 9940 Evergem, Belgium) and germinating seeds and once a week by fresh greens. Additionally, birds had access to a water bath 6 days a week. 12–16 days after hatching, individuals were given a black plastic ring with a unique identification number.

### Social treatments

The experimental treatment started at the beginning of the adolescent period (average age 41 days; range 36–45), when males were removed from their natal aviaries, and ended when the males were adult (average age 110 days, range: 104–114). This period was selected to ensure that the adolescent phase was covered until its end and all animals had reached sexual maturity [29]. Birds were either housed in juvenile pairs (one juvenile male and one juvenile female, 1 m/1f), in juvenile groups (three juvenile males and three juvenile females, 3 m/3 f), or in mixed-age groups (three juvenile males, three juvenile females, two adult males and two adult females, 5 m/5 f). Siblings were never assigned to the same group and randomized over the different social treatments. Adults assigned to the mixed-age groups originated from our lab stock and were pairs that had already successfully bred with each other. They were unfamiliar to the juveniles. In total, we formed 14 juvenile pairs (*n* = 14 males), six juvenile groups (*n* = 18 males) and six mixed-age groups (*n* = 18 males). However, seven males had to be removed from their groups due to disease or incorrect initial sex assignment. Although four of these males were replaced within the first week by males of similar age (mean age difference between all males: 4 days; range: 1–9 days), all males removed and all replacement males were excluded from statistical analyses. This resulted in a final sample size of 43 subjects, comprising 10 males from juvenile pairs, 18 males from juvenile groups and 15 males from mixed-age groups. Each pair or group was housed in a small aviary (100 × 200 × 200 cm). In total, six different indoor rooms were used, each room containing two to three aviaries with a juvenile pair, one aviary with a juvenile group and one aviary with a mixed-age group. Treatment groups had no visual contact. All rooms had a controlled 14 h light:10 h darkness photoperiod (lights on at 7:00 h), using fluorescent full-spectrum light tubes (Osram, Biolux, L58W/965), and received no natural daylight. Birds had ad libitum access to seed food and water. This was supplemented twice a week by germinating seeds and once a week by fresh greens and a water bath. In all aviaries there were two feeders to minimize potential differences in food competition between groups of different sizes. At the beginning of the treatment period, all birds were symmetrically ringed with a second black ring and two identical colour rings. Assignment of colour rings was randomized within sex and age categories, and balanced across social treatments.

**Fig. 1:**
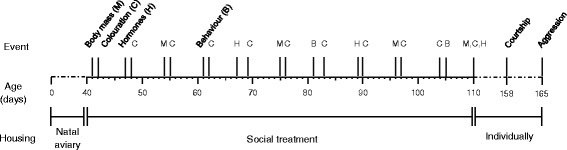
Time line of experimental events during adolescence and in adulthood. The following experimental events took place: M: Body mass measurement; C: Colouration scan; H: Blood sampling for hormone analysis; B: Behavioural observations; Courtship: Courtship song test; Aggression: Aggressiveness test. For details on the number of subjects tested see results

During adolescence, male social interactions and song were observed, T and CORT concentrations were measured, and the development of plumage colouration and weight was recorded. At the end of the adolescent period, males were individually housed and tested for their courtship and aggressive behaviour (Fig. 1).

### Behavioural observations

Behavioural observations of all 43 males were conducted three times during adolescence (Fig. 1) using focal animal sampling and continuous recording [52]. Each male was observed twice on a given observation day for a 10-min interval, resulting in a total of 1 h of focal animal observations. Observations were performed between 8:00 h and 13:00 h by the same experimenter (SB), and all subjects within the same treatment room were observed on the same day. Observation intervals were separated by a break of 1.5–2 h, during which the other males in the same room were observed. The order of observations in a session was randomized for the different social treatments within a room, but males within the same aviary were observed in succession before moving on to the next aviary. To minimize disturbance of the birds, the observer observed from behind a screen. At the start of each observation interval, the observer waited for 2 min to allow startled birds to resume normal activities.

The recorded behavioural patterns were defined as follows:

#### Plastic song

A series of different vocal elements in variable and poorly structured order [53–55].

#### Song

A song starts with two or more identical, so-called introductory elements [29, 56]. These are followed by a set of different vocal elements in a relatively fixed order [29, 56], which constitute a song motif. However, elements of a motif can be omitted or repeated, resulting in variable motif length [57, 58]. Several repeated motifs form a song [29].

Plastic song and song were defined to end when the male was silent for at least two seconds, when a new song was started by introductory elements, or when the male stopped singing while hopping to another perch.

If sequences of elements were not repeated, they were not recorded since they could not easily be classified either as plastic song or song or other vocalisations (for recordings and spectrograms of different types of song during development see e.g. [59].

#### Social interactions

We recorded the following behaviours that involved direct physical interactions between a focal male and another individual as well as whether the behaviours were initiated by or directed towards the focal male:

*Social exploration*: One individual holds or pulls the feet, tail, or feathers of another individual with its beak or grabs food from the other’s beak. *Preening*: One individual manipulates the feathers of another individual with its beak, performing a series of rapid movements (mostly on the head, but also on the back or sides). *Pecking:* One individual rapidly moves its beak towards another individual’s body and touches it. *Beak fighting:* Two individuals peck at each other with their beaks, head-on or laterally, while in an upright posture. *Chase:* One individual flies towards another, followed by the latter’s immediate displacement.

A social interaction ended when there was no more contact between individuals for two seconds or when the chased bird landed on a perch or the floor.

Since some behaviours were observed infrequently and not in all individuals, we summed up all social interactions for the analysis. Table 1 shows the frequencies of different behaviours recorded at each age.

**Table 1:** Experimental measures during adolescence and in adulthood

		Age at observation: 61 days						Age at observation: 81 days						Age at observation: 105 days					
a)	Behaviour/min	Directed from focal male to:				all	per	Directed from focal male to:				all	per	Directed from focal male to:				all	per
		juv ♀	juv ♂	ad ♀	ad ♂	partners	partner	juv ♀	juv ♂	ad ♀	ad ♂	partners	partner	juv ♀	juv ♂	ad ♀	ad ♂	partners	partner
Juvenile pairs	Social exploration	0.050				0.050	0.050	0.010				0.010	0.010	0.015				0.015	0.015
	Preening	0.035				0.035	0.035	0.010				0.010	0.010	0.035				0.035	0.035
	Pecking	0.010				0.010	0.010	0.025				0.025	0.025	0.045				0.045	0.045
	Beak fighting	0.005				0.005	0.005	0.000				0.000	0.000	0.010				0.010	0.010
	Chasing	0.000				0.000	0.000	0.000				0.000	0.000	0.000				0.000	0.000
Juvenile groups	Social exploration	0.042	0.022			0.064	0.013	0.039	0.019			0.058	0.012	0.042	0.028			0.070	0.014
	Preening	0.044	0.025			0.069	0.014	0.042	0.008			0.050	0.010	0.022	0.008			0.030	0.006
	Pecking	0.014	0.019			0.033	0.007	0.031	0.025			0.056	0.011	0.047	0.011			0.058	0.012
	Beak fighting	0.003	0.003			0.006	0.001	0.006	0.003			0.009	0.002	0.031	0.003			0.034	0.007
	Chasing	0.000	0.000			0.000	0.000	0.000	0.000			0.000	0.000	0.000	0.000			0.000	0.000
Mixed-age groups	Social exploration	0.040	0.020	0.037	0.013	0.110	0.012	0.020	0.010	0.000	0.013	0.043	0.005	0.010	0.007	0.013	0.003	0.033	0.004
	Preening	0.027	0.037	0.007	0.007	0.078	0.009	0.063	0.007	0.003	0.000	0.073	0.008	0.010	0.010	0.000	0.000	0.020	0.002
	Pecking	0.013	0.007	0.013	0.000	0.033	0.004	0.017	0.010	0.010	0.010	0.047	0.005	0.037	0.007	0.017	0.007	0.068	0.008
	Beak fighting	0.007	0.003	0.000	0.010	0.020	0.002	0.000	0.003	0.003	0.000	0.006	0.001	0.000	0.000	0.000	0.000	0.000	0.000
	Chasing	0.000	0.000	0.003	0.003	0.006	0.001	0.000	0.000	0.000	0.000	0.000	0.000	0.000	0.000	0.023	0.000	0.023	0.003
b)	Behaviour/min	Directed to focal male from:				all	per	Directed to focal male from:				all	per	Directed to focal male from:				all	per
		juv ♀	juv ♂	ad ♀	ad ♂	partners	partner	juv ♀	juv ♂	ad ♀	ad ♂	partners	partner	juv ♀	juv ♂	ad ♀	ad ♂	partners	partner
Juvenile pairs	Social exploration	0.085				0.085	0.085	0.025				0.025	0.025	0.020				0.020	0.020
	Preening	0.02				0.02	0.02	0.005				0.005	0.005	0.040				0.040	0.040
	Pecking	0.01				0.01	0.01	0.020				0.020	0.020	0.055				0.055	0.055
	Beak fighting	0.005				0.005	0.005	0.010				0.010	0.010	0.015				0.015	0.015
	Chasing	0.000				0.000	0.000	0.000				0.000	0.000	0.000				0.000	0.000
Juvenile groups	Social exploration	0.019	0.047			0.066	0.013	0.061	0.014			0.074	0.015	0.039	0.042			0.081	0.016
	Preening	0.039	0.011			0.050	0.010	0.047	0.047			0.094	0.019	0.044	0.008			0.052	0.010
	Pecking	0.017	0.008			0.025	0.005	0.033	0.039			0.072	0.014	0.036	0.014			0.050	0.010
	Beak fighting	0.014	0.006			0.020	0.004	0.000	0.000			0.000	0.000	0.011	0.000			0.011	0.002
	Chasing	0.000	0.000			0.000	0.000	0.003	0.000			0.003	0.001	0.000	--0.000			0.000	0.000
Mixed-age groups	Social exploration	0.050	0.047	0.013	0.003	0.113	0.013	0.060	0.020	0.003	0.000	0.083	0.009	0.037	0.010	0.003	0.000	0.050	0.006
	Preening	0.020	0.047	0.007	0.000	0.074	0.008	0.023	0.030	0.000	0.000	0.053	0.006	0.007	0.010	0.003	0.000	0.020	0.002
	Pecking	0.000	0.017	0.040	0.013	0.070	0.008	0.030	0.023	0.043	0.013	0.109	0.012	0.027	0.020	0.027	0.003	0.077	0.009
	Beak fighting	0.003	0.003	0.000	0.007	0.013	0.001	0.000	0.000	0.003	0.000	0.003	0.001	0.007	0.007	0.003	0.000	0.017	0.002
	Chasing	0.000	0.000	0.000	0.000	0.000	0.000	0.000	0.000	0.000	0.000	0.000	0.000	0.000	0.000	0.000	0.000	0.000	0.000

Given are the mean age of subjects and the number of subjects tested at each measure. For details see materials and methods. Frequencies of different behaviours towards different interaction partners recorded at each observation during adolescence. Behaviours recorded a) initiated and b) received by focal males were social exploration, preening, pecking, beak fighting and chasing. Interaction partners: juv. ♀: juvenile female; juv. ♂: juvenile male;ad. ♀: adult female; ad. ♂: adult male

### Hormones

Blood samples were taken three times during adolescence (Fig. 1) to analyse T and CORT levels during development. Sample sizes for T differed between days because T was only measured if at least 15 μl plasma was still available after CORT determination. Sample sizes for CORT analyses differed as not all samples could be obtained within 3 min after entering a treatment room [60, 61]. It was not possible to repeat collection of these samples because that would have caused an unequal disturbance of different birds, thereby possibly affecting other experimental variables. Moreover, welfare considerations would have only allowed renewed sampling 1 week later. Blood samples were always collected between 11:00 h and 12:30 h to minimize circadian changes in T and CORT levels [62, 63]. Each day, only males from one social treatment per room were sampled so that samples were taken over three successive days. Blood sampling was randomised for the social treatments across treatment rooms at each sampling session. Furthermore, we randomised the order of blood samples taken from males in different social treatments in the same room across sampling sessions. Birds were caught from the aviaries with a net and blood was collected in heparinised capillaries after puncturing the ulnar vein with a hypodermic needle. Capillaries were directly stored on ice. After a maximum of 1 h, plasma was separated by centrifugation (5000 rpm for 10 min) and frozen at −20 °C until further processing.

T concentrations in plasma were determined in duplicate by enzyme immunoassay kits (DES 6622, Demeditec Diagnostics GmbH, Kiel, Germany) and then averaged. The antiserum used cross-reacted with relevant steroids as follows: testosterone 100%, 5α-dihydrotestosterone 23.3%, androstenedione 1.6%, and all other tested steroids < 0.1%. The intra-assay coefficient of variation (CV) was 7.5% and the inter-assay CV was 9.1%. T concentrations were not detectable in 42 out of 150 samples. These 42 samples were assigned a value of zero for the analysis, to be conservative (assay sensitivity is 2.2 pg/ml, and samples had to be diluted between 3 and 44 times because variable amounts of blood were obtained during sampling). However, excluding these samples did not change the significance of the results or interpretation.

CORT concentrations were determined using corticosterone enzyme immunoassay kits (500655, Cayman Chemical, Michigan, USA) and were detectable in all samples. They were measured initially in duplicate, but if the % CV of the first duplicate measurement was higher than 15% and there was still sufficient plasma, they were measured in quadruplicate and subsequently averaged over all measurements. The antiserum used cross-reacted with relevant steroids as follows: corticosterone 100%, 11-dehydrocorticosterone 11%, 11-deoxycorticosterone 7%, progesterone 0.31%, cortisol 0.17%, and all other tested steroids < 0.1%. The intra-assay CV was 10.1 and the inter-assay CV was 10.6%.

### Colouration

**Fig. 2:**
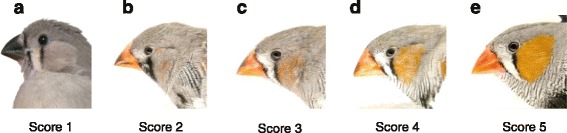
Scores for the development of the male-typical colouration. Traits scored were beak, cheek patches and breast stripes. The pictures shown demonstrate cheek patch development as an example. **a** Score 1 = no colouration of cheek patch; **b** Score 2 = less than 1/3rd of cheek patch coloured; **c** Score 3 = 1/3rd – 2/3rd of cheek patch coloured; **d** Score 4 = more than 2/3rd of cheek patch coloured; **e** Score 5 = complete colouration of cheek patch

Males were visually scored once a week (Fig. 1) from outside the aviaries to quantify the development of the adult male colouration [29, 45]. Traits scored included beak, cheek patches and breast stripes, with each trait scored separately for the left and the right body half of each male. Scores given ranged from 1 to 5 as follows: 1 (no colouration), 2 (less than 1/3^rd^ coloured), 3 (between 1/3^rd^ and 2/3^rd^ coloured), 4 (more than 2/3^rd^ coloured) and 5 (fully coloured) (Fig. 2). The separate scores on a given day were averaged for each subject for statistical analyses. The first colour score was not obtained for about half of the subjects due to time constraints when starting the experiment and the final score was not taken for one animal that was ill.

### Body mass

During the treatment period, males were weighed five times (Fig. 1). For practical reasons, the first weighing took place between 10:00 h and 13:00 h of the day on which treatment groups were assigned. Subsequent weights were taken between 13:00 h and 18:00 h and within 2 h for all males housed in the same room. The first two weights were taken for only about half of the birds due to time constraints at the start of the experiment.

### Adult housing

After the end of the social treatments males were housed individually in cages (30 cm high × 40 cm wide × 40 cm deep). We used the same protocol as previous experiments [38, 43, 64] to ensure that the design was comparable and that any long-term behavioural modifications could solely be explained by differences in social experiences during adolescence. Males were housed in the same room where they had hatched and had no visual, but auditory contact with each other and other birds in the room. Food and water was provided in the same manner as during the social treatment phase. On the transfer day, all rings were removed except the black numbered ring.

### Courtship song test

Female-directed song (see definition of *song*) was quantified by presenting an unfamiliar female in a cage attached to the front of a male’s cage. For each male, one of 16 different stimulus females was used whose presentation was randomized across males of different social treatments. We recorded the latency to start singing and the number of motifs produced in 10 min. Males that did not sing were assigned the maximum latency of 600 s and a motif rate of zero (6 pair-housed males, 3 juvenile group-housed males, and 6 mixed-age-group-housed males, a non-significant difference; *χ* ^2^ = 3.4, df = 2, *p* = 0.2). Courtship song tests were performed between 9:00 h and 11:30 h in the males’ home cages. The average age of males at courtship testing was 158 days (range: 152–161 days). One juvenile group male died shortly after the end of the social treatment phase, therefore only 42 males were included in the courtship song test.

### Aggressiveness test

The aggressiveness test was performed 5–10 days after the courtship test for each individual (average age 165 days; range 161–169 days). Male-male competition was tested in dyads by placing two males in a cage together with an unfamiliar female. Males were pseudo-randomly assigned to dyads of males from juvenile pairs and males from juvenile groups (*n* = 4), dyads of males from juvenile pairs and males from mixed-age groups (*n* = 5), and dyads of males from juvenile groups and males from mixed-age groups (*n* = 7). The remaining males were paired with replacement males or similar aged males that had remained in the natal breeding aviary. Initially, we included these males in the analysis but later decided against it because their number was too low for statistical analysis and their rearing conditions too different. Therefore the final sample size for the aggressiveness test was *n* = 32, with *n* = 9 for males from juvenile pairs, *n* = 11 for males from juvenile groups and *n* = 12 for males from mixed-age groups. In total, every male was tested once, in only one of the possible test combinations and brothers were never tested against each other.

A day before testing, the two males of a dyad were placed into a cage (30 cm high × 80 cm wide × 40 cm deep) which was divided by a non-transparent partitioning wall into two equally sized halves. Each half of the cage was equipped with two perches, a feeding station and a water dispenser. Males of a dyad were ringed with black or pink colour rings on both legs, with colours being equally allocated to males of different social treatments. To control for potential side effects during testing, initial placement of males into the right or left side of the cage was balanced across social treatments. Tests started the next morning by removing the partitioning wall between males and releasing a female into the cage. Subsequently, the number of chases males performed towards each other was recorded as a measure of aggressiveness. The 16 stimulus females used for the aggressiveness test were the same ones that had been used for the courtship song test, but they were always unfamiliar to both males of a tested dyad. Each test lasted 1 h. Test sessions started between 8:00 h and 9:00 h and ended between 12:00 h and 13:00 h, as four male dyads were tested consecutively. Aggression tests were performed in the males’ standard housing room.

### Data analysis

Some data were transformed to achieve equal variances between social treatment groups and normally distributed residuals. Variances and distributions were assessed visually using variance plots, histograms of residuals and Q-Q plots. A square root transformation was used for plastic song, song motifs and chases (n/min). A log_10_ transformation was used for T and CORT concentrations (ng/ml), after adding a value of one to each measure. Body mass, colouration, and the courtship song latency met the criteria of variance homogeneity and normally distributed residuals and were thus analysed using the untransformed values. Social interactions (n/min) during development were Poisson distributed and analysed by a generalised linear mixed model (GLMM) using a Poisson distribution. All other data were analysed by linear mixed models (LMM), assuming a normal distribution.

Data were analysed in R 2.13.1 by mixed effects models using a maximum likelihood approach (package lme4). Significances of effects were calculated using likelihood ratio tests. Effects with a *p* > 0.1 were removed stepwise from the model. The highest order interactions were always tested first, followed by lower order interactions, until the final model was obtained. Whenever higher order interactions were significant, all lower order interactions remained in the model. Main effects of social treatment and age were always kept in the model. Subsequent pairwise comparisons between two experimental treatments were conducted using Sidak adjustments to account for multiple testing.

The analyses included a random effect of the experimental aviary (“treatment group ID”), a random effect of the male’s family (“nest ID”), and a random effect of the male’s identity (“male ID”) for data with multiple measures of each male. “Nest ID” and “male ID” were kept in all models to control for non-independence of multiple measures from the same male and from brothers allocated to different treatment groups. A random effect of “treatment group ID” was removed from the models if it was clearly not statistically significant (*p* > 0.2).

To analyse the differences in development between the three social treatments, the effects of treatment (“social treatment”), age at testing (“age”) and their interaction were tested. Since the change with age in most traits was non-linear, we included the effects of higher order polynomials of “age”, which is a frequently used method to model non-linear slopes in a mixed-modelling framework [65]. We included up to the 5^th^ polynomial of age if significant. As the effect of the social treatment is likely to be weaker at the start of the experiment and might affect the birds more at certain ages than others, we expected to find significant interactions between treatment and age. In most cases, an interaction with a polynomial of age was significant. In some cases, only the main effect of the polynomial of age was significant and hence stayed in the model (for corticosterone: age^3^; body mass: age^3^; colouration: age^5^).

Adult behaviour in the courtship song test was analysed with “social treatment” as the only fixed factor. Adult behaviour in the aggressiveness test was analysed with “social treatment” and type of opponent (“opponent treatment”) as fixed factors. Since males were never tested with an opponent from their own social treatment, post-hoc comparisons of experimental groups could not be performed on the interaction of “social treatment” and “opponent”. Therefore, we only tested for a main effect of opponent by choosing one experimental group and “opponent” as the only fixed factor. Analyses for an effect of opponent were conducted using linear models (LM), as there were no random effects. Since a normal distribution could not be achieved by any transformation of motif rate or rate of chases, these data were also analysed non-parametrically using Kruskal-Wallis tests and pairwise Wilcoxon tests. This did not change the significance of any result, therefore only the parametric statistics are presented in the figures and text.

Graphs show means ± standard error (SE) estimated with “social treatment”, “age”, and “opponent treatment“as categorical factors, including the random effects. For models including age as a factor, graphs also show the prediction lines from the final models. The significance level α was set at *p* < 0.05.

## Results

### Social interactions

**Fig. 3:**
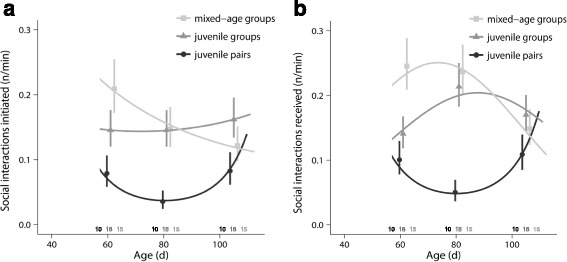
Social interactions during adolescence. Males reared in groups **a ** initiated more social interactions than males from juvenile pairs and **b﻿ **differed from them with regard to how received social interactions changed as they matured. Furthermore, group males differed from each other with regard to how the frequency of interactions **a ** initiated and **b ** received changed during development. For details, see text. Shown are means ± SE for each age and lines from the model with the best fit. Sample sizes are shown directly above the x-axis

The interaction of treatment and age had a significant effect on the frequency of social interactions initiated by the focal males (GLMM: social treatment x age: *χ* ^2^ = 7.0, df = 2, *p* = 0.03; Fig. 3a) and on the frequency of social interactions directed by other birds towards the focal males (GLMM: social treatment x (age + age^2^): *χ* ^2^ = 19.9, df = 4, *p* < 0.001; Fig. 3b).

When comparing pairs of experimental groups, males from mixed-age groups and males from juvenile groups differed with regard to how the social interactions they initiated changed as they matured (GLMM: social treatment x age: *χ* ^2^ = 6.8, df = 1, *p* < 0.03). The same was found for the social interactions they received (GLMM: social treatment x (age + age^2^): *χ* ^2^ = 8.4, df = 1, *p* < 0.05). Mixed-age group males had most interactions during early adolescence, but these decreased towards late adolescence. In juvenile groups, social interactions slightly increased from early to late adolescence. Males from both group conditions also initiated on average more social interactions than males from juvenile pairs (GLMM: social treatment: *χ* ^2^ > 7.2, df = 1, *p* < 0.02; to test whether there was an overall difference in social interactions we removed the effect of the interaction of social treatment and age for this analysis). Furthermore, males from both group treatments differed significantly from males in juvenile pairs with regard to how the social interactions directed towards them changed as they matured (GLMM: social treatment x (age + age^2^): *χ* ^2^ > 9.7, df = 1, *p* < 0.02). In juvenile pairs, social interactions directed towards males initially decreased, but then increased again. The opposite pattern was seen in group-housed males.

### Plastic song and song

**Fig. 4:**
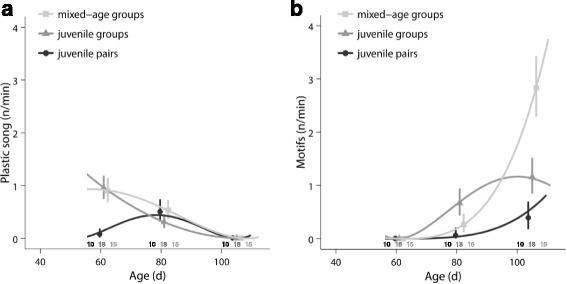
Plastic song and song during adolescence. **a** Group males showed higher rates of plastic song than pair males during early adolescence. **b** Mixed-age group males showed a higher increase in song motif rate than juvenile group males and juvenile pair males in late adolescence. For details, see text. Shown are means ± SE for each age and lines from the model with the best fit. Sample sizes are shown directly above the x-axis

The social environment experienced during adolescence significantly influenced how male plastic song (Fig. 4a) and song (Fig. 4b) changed during development (LMM for song: social treatment x (age + age^2^): *χ* ^2^ = 16.7, df = 4, *p* = 0.002; LMM for plastic song: social treatment x (age + age^2^): *χ* ^2^ = 17.5, df = 4, *p* = 0.002).

Males from mixed-age groups differed significantly from males reared in juvenile pairs in the development of plastic song (LMM: social treatment x (age + age^2^): *χ* ^2^ = 10.4, df = 2, *p* < 0.02). The same was true for males from juvenile groups (LMM: social treatment x (age + age^2^): *χ* ^2^ = 14.3, df = 2, *p* = 0.002). There was no significant difference between the group treatments in plastic song development (LMM: social treatment x (age + age^2^): *χ* ^2^ = 1.3, df = 2, *p* > 0.9). Group-reared males showed high rates of plastic song during early adolescence when pair-reared males produced almost no plastic song. The frequency of plastic song was similar during late adolescence when pair-reared males started producing plastic song.

The change of song with age differed significantly between males reared in mixed-age groups and those reared in juvenile groups (LMM: social treatment x (age + age^2^): *χ* ^2^ = 11.9, df = 2, *p* < 0.01). Furthermore, the change of song with age differed significantly between males reared in mixed-age groups and males reared in juvenile pairs (LMM: social treatment x (age + age^2^): *χ* ^2^ = 9.6, df = 2, *p* = 0.024). Juvenile group males and juvenile pair males did not differ significantly in song development (LMM: social treatment x (age + age^2^): *χ* ^2^ = 4.7, df = 2, *p* = 0.26). Males from mixed-age groups increased song motif rate most strongly towards late adolescence. Males from juvenile groups showed a more constant moderate increase in motif rate from early to late adolescence. Finally, males from pairs showed a slight increase in motif rate only in late adolescence.

### Hormones

#### Testosterone

**Fig. 5:**
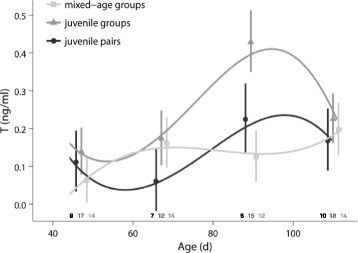
Testosterone levels during adolescence. Juvenile group males had a pronounced peak in testosterone (T) in late adolescence, which was missing in males from mixed-age groups. For details, see text. Shown are means ± SE for each age and lines from the model with the best fit. Sample sizes are shown directly above the x-axis

There was a significant effect of the interaction between social treatment and the cubic part of the curve fit (LMM: social treatment x age^3^: *χ* ^2^ = 6.8, df = 2, *p* = 0.03; Fig. 5), indicating that the social environment during adolescence influenced plasma T profiles. However, the difference in the overall temporal profile in T did not reach significance (LMM: social treatment x (age + age^2^ + age^3^): *χ* ^2^ = 10.0, df = 6, *p* = 0.1). T profiles of males from mixed-age groups and males from juvenile groups differed significantly in the cubic part of the curve fit (LMM: social treatment x age^3^: *χ* ^2^ = 6.1, df = 1, *p* = 0.04). There was no significant difference in T profiles (LMM: social treatment x age^3^: *χ* ^2^ < 4.5, df = 1, *p* > 0.1) or average T levels (LMM: social treatment: *χ* ^2^ < 1.9, df =1, *p* > 0.1) between the other treatments. Males from juvenile groups showed a pronounced peak in T in late adolescence. In mixed-age groups T levels increased only slightly in early adolescence and again in late adolescence and there was no peak at any age. Males from juvenile pairs showed a slight decrease in T in early adolescence and a moderate increase in late adolescence.

#### Corticosterone

**Fig. 6:**
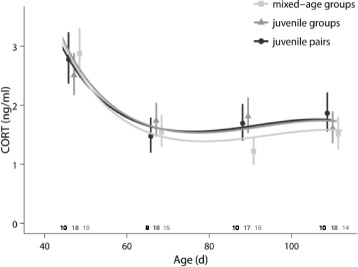
Corticosterone levels during adolescence. Corticosterone (CORT) profiles of males during adolescence did not differ between treatments. For details, see text. Shown are means ± SE for each age and lines from the model with the best fit. Sample sizes are shown directly above the x-axis

Plasma CORT profiles were not affected by the social environment during adolescence (social treatment x age: *χ* ^2^ = 2.4, df = 2, *p* = 0.3; social treatment: *χ* ^2^ = 0.5, df = 2, *p* = 0.8; Fig. 6). All males showed a significant decline in plasma CORT with age (age + age^2^ + age ^3^: *χ* ^2^ = 38.6, df = 3, *p* < 0.001).

### Colouration

**Fig. 7:**
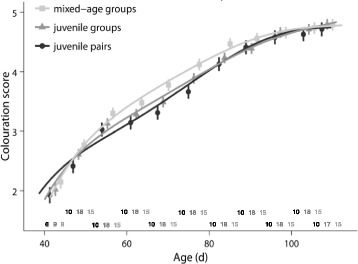
Plumage colouration during adolescence. Males from mixed-age groups developed the adult colouration significantly faster than males from juvenile pairs and males from juvenile groups. For details, see text. Shown are means ± SE for each age and lines from the model with the best fit. Sample sizes are shown directly above the x-axis

The development of the male-typical colouration during adolescence differed significantly between males from different social rearing environments (LMM: social treatment x (age + age^2^ + age^3^): *χ* ^2^ = 23.7, df = 6, *p* < 0.001; Fig. 7). Males from mixed-age groups developed the characteristics of the adult male plumage significantly faster than males from juvenile pairs (LMM: social treatment x (age + age^2^ + age^3^): *χ* ^2^ = 16.4, df = 3, *p* < 0.01). In addition, males from mixed-age groups developed the adult plumage traits significantly faster than males from juvenile groups (LMM: social treatment x (age + age^2^ + age^3^): *χ* ^2^ = 14.1, df = 3, *p* < 0.01). Juvenile group males and juvenile pair males did not differ in the development of plumage colouration (LMM: social treatment x (age + age^2^ + age^3^): *χ* ^2^ = 4.5, df = 3, *p* = 0.5).

### Body mass

**Fig. 8:**
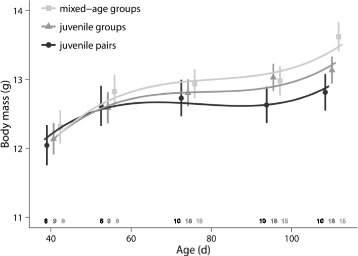
Body mass during adolescence. Males from mixed-age groups tended to gain more weight during adolescence than males from juvenile pairs. For details, see text. Shown are means ± SE for each age and lines from the model with the best fit. Sample sizes are shown directly above the x-axis

Although statistically not significant, the increase in weight with age during adolescence tended to differ between males from different social rearing environments (LMM: social treatment x age: *χ* ^2^ = 4.8, df = 2, *p* = 0.09; Fig. 8). Males that grew up in mixed-age groups tended to gain more weight than males that grew up in juvenile pairs, especially during late adolescence (LMM: social treatment x age: *χ* ^2^ = 5.1, df = 1, *p* = 0.07). There was no difference between the other treatments (LMM: social treatment x age: *χ* ^2^ < 2.3, df = 1, *p* > 0.3).

### Courtship song test

**Fig. 9:**
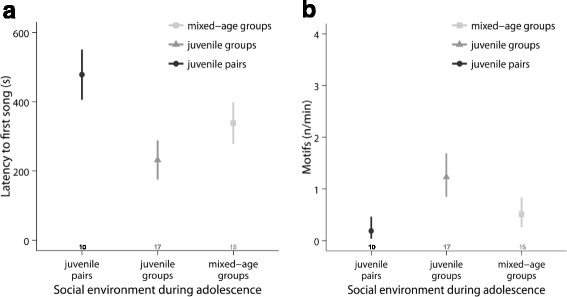
Courtship singing in adulthood. Males from juvenile groups **a** started to sing significantly faster than males from juvenile pairs and **b** showed a tendency to sing with a higher motif rate than males from juvenile pairs. For details, see text. Shown are means ± SE for each age. Sample sizes are shown directly above the x-axis

In the courtship song test in adulthood, there was a significant difference in the latency to start the first song between males from different social-rearing environments (LMM: social treatment: *χ* ^2^ = 8.9, df = 2, *p* = 0.01; Fig. 9a). In addition, motif rates tended to differ between social treatments (LMM: social treatment: *χ* ^2^ = 5.8, df = 2, *p* = 0.06; Fig. 9b). Males from juvenile groups started to sing significantly faster than males from juvenile pairs (LMM: social treatment: *χ* ^2^ = 9.3, df = 1, *p* = 0.007). Furthermore, they showed a tendency to sing more motifs per minute than pair males. (LMM: social treatment: *χ* ^2^ = 5.8, df = 1, *p* = 0.05). The other treatments did not differ significantly in singing latency (LMM: social treatment: *χ* ^2^ < 3.0, df = 1, *p* > 0.24) or motif rate (LMM: social treatment *χ* ^2^ < 2.7, df = 1, *p* > 0.27).

### Aggressiveness test

**Fig. 10:**
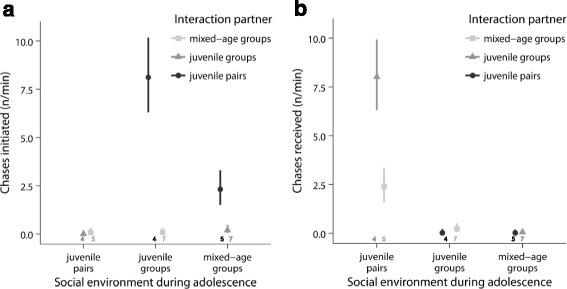
Aggressiveness in adulthood. **a** Males from groups initiated more chases when their opponent was from a juvenile pair than when he was from the other group treatment, whereas males from juvenile pairs directed few chases towards males from either group treatment. **b** Males from groups received fewer chases than males from juvenile pairs from all interaction partners. For details, see text. Shown are means ± SE for each age. Sample sizes are shown directly above the x-axis

There was a significant effect of the interaction between social treatment and opponent treatment on the rate of chases initiated by the focal males in adulthood (LMM: social treatment x opponent: *χ* ^2^ = 7.1, df = 1, *p* < 0.008; Fig. 10a). The same was true for the rate of chases directed by other birds towards the focal males (LMM: social treatment x opponent: *χ* ^2^ = 8.3, df = 1, *p* < 0.004; Fig. 10b). Post-hoc tests revealed that males from juvenile groups (LM: t-value = 6.0, *p* < 0.001) and males from mixed-age groups (LM: t-value = 2.5, *p* = 0.03) showed a significantly higher rate of aggression towards males from juvenile pairs than towards males from the other group treatment. In contrast, males from juvenile pairs showed similarly low rates of aggression towards both males from juvenile groups and males from mixed-age groups (LM: t-value = 1.5, *p* = 0.19). Further post-hoc tests on the aggression directed towards focal males confirmed that males reared in juvenile pairs received most aggression and males reared in groups received least aggression (Fig. 10b, analysis not shown).

## Discussion

In recent years, more and more studies have found long-lasting effects of the adolescent social environment on adult behaviour, yet we still know very little about the underlying behavioural and physiological mechanisms. We describe for the first time in zebra finches how the size and age composition of social groups during adolescence affect social interactions, song development, plumage colouration and T concentrations with long-lasting consequences for adult courtship and aggressive behaviour. Our results suggest that the effects of the social environment on adult behaviour may be mediated by differences in behavioural and physiological maturation.

### Maturation and adult behaviour

Group housing during adolescence enhanced the frequency of social interactions, song development, plumage colouration and the frequencies of courtship and aggressive behaviour in adulthood. Although some effects were more pronounced in juvenile groups, others were stronger in mixed-age groups. Earlier studies on zebra finches found that social isolation delayed song development [46, 47] and plumage maturation [45]. Our results suggest that this is not an artefact of extreme social deprivation, but that male zebra finches can mature more rapidly at higher social densities. Rapid maturation at high densities may be beneficial if higher densities indicate a larger variety of potential partners or, more generally, a high quality environment that is more suitable for reproduction. Since zebra finches are monogamous, adult males do not monopolise females at high densities. In the polygynous guinea pig by contrast, males reared at high densities are thought to follow a queuing strategy associated with low aggression and courtship to avoid competition with adult males [2]. In the wild, maturation in zebra finches is also affected by the early environment, as males born early in the season develop adult plumage more rapidly and can breed at a much younger age than males born late in the season, the latter delaying plumage maturation and breeding until the next season [29]. Even though zebra finches are opportunistic breeders with early sexual maturation, they nevertheless appear to be able to benefit from adjusting reproductive investment to social and ecological conditions [66, 67].

Group-housing not only stimulated maturation, but also increased adult courtship behaviour, which may be beneficial when reproductive opportunities increase. In zebra finches, females prefer e.g. males with a high motif rate [68]. In addition, group housing increased aggressiveness, but only towards pair-housed males. This suggests that the appearance or behaviour of the opponent is crucial in stimulating higher aggressiveness. Similar effects have been described in male Syrian golden hamsters where the experience of losing in agonistic interactions with adult males can result in enhanced levels of aggression during adulthood [69], especially when confronted with inferior opponents [70]. This might explain why males reared only with a juvenile female received most aggression: males reared in pairs may trigger more aggression in group-reared males as they have not learned to display appropriate social behaviour towards same sex opponents and hence appear less socially skilled or inferior to the more competent group reared males. Importantly, the effects on adult courtship and aggression contrasted with earlier studies on the influence of the adolescent social environment on adult behaviour in zebra finches [38, 43], which will be discussed further below.

### Differences between groups with and without adults

Some effects of group housing on behaviour and physiology differed between juvenile groups and mixed-age groups. We cannot disentangle whether these effects are due to group composition, group size or housing density because mixed-age groups were larger than juvenile groups and aviaries had the same size so that less space was available per individual. Also, males in mixed-age groups had nearly twice as many interaction partners than those in juvenile groups, but not twice as many social interactions, and during late adolescence they had even fewer social interactions. We therefore do not know whether the observed effects are due to an increase in social interactions, due to a larger number of interaction partners or possibly even due to a reduction in social interactions with each individual in the group. However, we presume that it is more likely that the observed differences are due to the presence of adults rather than group size or density. High densities often have negative effects on body mass [71], but in this experiment and previous studies in our lab [38], there were no significant differences in body mass between experimental groups. In contrast, the presence of adults during development has been found to influence the frequency and type of interactions juveniles’ experience in several species, with species-specific consequences for their maturation and adult behaviour [1, 5, 14, 37, 72]. Interestingly, the presence of adults can have different effects depending on the age during which adolescents interact with them [4] and the context in which they are tested as adults [5, 70]. Thus, the presence of adults can both stimulate and inhibit sexual maturation of young, which may explain some of our results. If we split the experimental phase that lasted from shortly after nutritional independence to early adulthood (day 41 to day 110) into two periods, we can distinguish between early adolescence (~day 41–75) and late adolescence (~day 76–110). We choose this division because sexual maturity is attained between day 60–90 in zebra finches [29], ~day 75 being the median age. Early adolescence thereby represents the time period before the first reproductive event, while late adolescence represents the period during which all birds reach sexual maturity. During early adolescence, the presence of adults might have a positive effect because young zebra finches and other birds are e.g. attracted to adult males to learn song [53, 73–77]. Therefore in our study, the presence of adults might have increased social interactions, thereby stimulating plumage maturation in early adolescence. As a result, mixed-age group males might have moulted into the adult plumage faster than juvenile pair males or juvenile group males.

In contrast, during late adolescence when males are close to sexual maturity, interactions with adults may occur in a context of competition and reproduction. Zebra finches are monogamous and males and females guard their mates against approaches by same-sex rivals (Zann 1996). Especially in juveniles the frequency of courtship attempts towards mated individuals is high (Zann 1996). We suggest that through the behavioural feedback of interaction partners juveniles learn about the discrimination of interaction partners that are already paired or still available. Unfortunately, we did not have enough behavioural data to analyse sexual and aggressive behaviour with different interaction partners during adolescence. However, interactions with adults can accelerate and improve sexual behaviour of young during development and adulthood in other species [4, 37, 72, 78]. Juvenile males in mixed-age groups may have had more opportunities to learn and fine-tune their behaviour, which could explain the increase in singing rate during late adolescence in mixed-age group males. However, in adulthood, courtship singing was highest in juvenile group males, not in mixed-age group males. This may be because in mixed-age groups juvenile males have learned that they are less successful in courtship due to the presence of more attractive and competitive adult males and may therefore have been inhibited by their previous experiences. Zebra finch males that are less successful in pairing with female conspecifics as juveniles also have reduced pairing success later in life [3].

While the frequency of social interactions in mixed-age group males decreased from early to late adolescence, the opposite was seen in juvenile group males. We suggest that males in juvenile groups initially interacted less due to their smaller group sizes or the absence of adults during early adolescence, but they increased social interactions when approaching sexual maturity as they were not inhibited by older, more experienced males.

In conclusion, social interactions, song and plumage developed differently in groups with adults than in those without adults, but there was little evidence of differences in adult behaviour. This suggests that the most striking, long-lasting effects of the social environment during adolescence on adult courtship and aggression observed in zebra finches in this and earlier studies [38, 43] may not primarily depend upon the age structure of groups.

### The role of endocrine changes

The observed differences in plumage maturation and behaviour may partly be due to endocrine changes. During early adolescence, plumage maturation was enhanced in groups with adults. At that age there were no differences in T levels, therefore we have to consider other hormones that are involved in social interactions and regulate plumage colouration [79–83]. In many avian species, estradiol and luteinizing hormone (LH) are important regulators of plumage colouration [79, 82] and thus should be measured in future studies. Although LH stimulates the secretion of T from the gonads, the gonads may still be insensitive to LH during early adolescence [84] which could explain why we found little evidence for differences in T levels. Plumage development may also be related to nutritional differences [85], although the higher weight gain of mixed-age group males compared to juvenile pair males during adolescence did not reach significance. Moreover, it has been shown that interactions between hormones and nutrition result in differences in plumage colouration, as hormones affect the deposition of pigments from food items in the feathers [79, 82].

In contrast to plumage differences, some behavioural differences between males during development and in adulthood may directly reflect differences in T levels. Juvenile group males did experience a T peak in late adolescence, as reported earlier for this stage of life in socially housed zebra finch males [48]. This peak was absent in mixed-age group males, most likely due to an inhibiting effect of the adults present in the groups (e.g. 86, 87]). As the T peak normally occurring in late adolescence coincides with a sensitive period for song learning [48] and the absence of T or an excess of T impairs the crystallization of song in zebra finches and other songbirds to its final stable form [88, 89], this might explain why mixed-age group males still increased song rates during that time. Their song rates (ca. 3 motifs/min) are more than tenfold higher than song rates previously observed in captivity or in the wild (less than 0.2 motifs/min, [37, 90], and more similar to song rates seen during the first 10–15 min when males first encounter unfamiliar females (ca. 1–4 motifs/min, [38, 43]). In contrast, elevated T in juvenile groups may have inhibited further changes in song during late adolescence.

Although T concentrations and song development during adolescence differed significantly between juvenile groups and mixed-age groups, adult courtship and aggression did not differ significantly between them. At the same time, T concentrations during adolescence did not differ significantly between juvenile pair males and juvenile or mixed-age group males, but adult courtship was significantly lower in juvenile pair males compared to juvenile group males. Moreover, adult aggression was significantly lower in juvenile pair males compared to males from juvenile and mixed-age groups. Therefore, there is no evidence that differences in T during adolescence affected adult behaviour. Instead, other hormones or social learning during adolescence may be more important for shaping adult behaviour during this period [76, 88, 89].

In addition to organisational effects of T, long-lasting effects of the early social environment are often explained by effects on CORT levels [91, 92]. Zebra finches are highly social birds and social isolation affects CORT levels [50, 93]. Social isolation of zebra finch males during adolescence also results in delayed plumage maturation [45] and song development [46, 47] compared to socially reared controls, which could be due to elevated CORT levels caused by social stress. However, we found no differences in CORT profiles between males in the different housing conditions, suggesting there were neither differences in social stress nor an effect of CORT on the behavioural differences we found. This is in line with an earlier study that found no effect of social density on CORT levels during development [71]. The decrease of CORT during early adolescence found in all social conditions in the present study could be attributed to an initial stress response when removed from the familiar environment of the natal aviaries and subsequent habituation to the new social environments. Habituation to new environments has been shown to occur within 30 min in zebra finches [93], but it is not known whether the same is true for a novel social environment. Since CORT levels in our study were high when first measured several days after introduction to the social treatments, this may reflect a stress response when adjusting to the new social environment or a maturational change of CORT profiles during development [71], although other studies suggest that basal CORT levels do not vary with age in zebra finches [19].

### Comparison with earlier experiments

In contrast to our expectations and earlier results [38, 43], adult courtship and aggression was elevated in males reared in groups compared to males reared in pairs, although this was only statistically significant for courtship in males from juvenile groups and for aggression in males from mixed-age groups. In previous studies it was suggested that males may court and compete less at high densities to prevent costly competition [43]. Similar behavioural differences were seen in guinea pigs and wild cavies reared under increased or instable social densities, which have been interpreted as an adaptive “queuing strategy” or “behavioural camouflage strategy” [2, 7, 32]. The results from the current study show that this interpretation has to be re-evaluated for zebra finches. Interestingly, adult song rates of pair-housed males in the current study were about three times lower than in the previous studies, whereas song rates of males from juvenile groups were similar [43]. Also, the courtship latency was higher and the level of aggressiveness shown towards opponents was lower in males reared in pairs in the current study compared to previous studies, whereas courtship latencies and rates of aggression of males reared in groups differed less. In the following, we discuss differences between the present study and earlier experiments which may have especially affected males housed in pairs and therefore explain the discrepancy in results.

First, we kept males in rooms with constant photoperiod during adolescence, whereas previously males experienced natural variation in photoperiod. Photoperiod affects morphological, physiological and behavioural development, as well as adult behaviour in many species [e.g. 94, 95], including zebra finches [29, 66, 96]. Effects of the social environment might thus depend on photoperiod and thereby explain the contrasting effects between our study and the previous ones. Moreover, studies in zebra finches suggest that photoperiodic effects may be masked by stimulatory effects of food abundance and social interactions [67, 97]. The constant photoperiod in our study might therefore have more strongly affected males housed in pairs because they experienced less social stimulation.

A second potential explanation for the differing results from previous research is a difference in acoustic stimulation during development. In this study males of different treatments were housed in the same indoor rooms, while males in previous studies were spatially farther apart and no groups containing both adults and juveniles were nearby. Since acoustic stimulation can modify adult behaviour and development [98], we suggest that males housed in pairs may have been most affected by the vocal interactions of adults and juveniles in adjacent mixed-aged groups.

Finally, male aggression was previously observed in triads [38] or in small groups [43]. If aggression of pair-reared males depends on social density, this may explain why we found low aggression when testing males in dyads. In previous studies, pair-reared males were also more aggressive than group-reared males when later tested in dyads [99], but this may have been a consequence of winner-loser effects during the first tests.

## Conclusion

Our study shows that the social environment during adolescence affects social interactions, plumage maturation and song development of male zebra finches with long-lasting consequences for adult courtship and aggressive behaviour. Contrary to our initial hypotheses, we do not find elevated T and CORT levels in groups as described for guinea pigs. The difference seen in T levels between males in juvenile groups and mixed-age groups may be linked to differences in song development, but not in plumage maturation or to the long-lasting effects on behaviour, and CORT is unlikely to play any role. The effect of the social environment during adolescence on other hormones should therefore be considered. The intriguing differences between our study and previous ones point to the multitude of factors shaping development. Future studies should therefore investigate how social factors interact with ecological conditions.

## Abbreviations

CORT: Corticosterone
CV: Coefficient of variation
GLMM: Generalised linear mixed model
HPA axis: Hypothalamo-pituitary-adrenal axis
HPG axis: Hypothalamo-pituitary-gonadal axis
LH: Luteinizing hormone
LMM: Linear mixed model
SE: Standard error
T: Testosterone
